# Risk Assessment of Osteoarthritis Among Geriatric Population in Perambalur District Using the Western Ontario and McMaster Universities Arthritis Index and Katz Index of Independence in Activities of Daily Living: A Cross-Sectional Study

**DOI:** 10.7759/cureus.39323

**Published:** 2023-05-22

**Authors:** Ramkumar Sundaram, Vijayalakshmi Srinivasan, Shagirunisha Rizvana, Kayalvizhi Saraboji, Kishore Kannan Muthusamy, Indhumathi Murugan, Keerthi Priya Karunanithi

**Affiliations:** 1 Department of Community Medicine, Dhanalakshmi Srinivasan Medical College & Hospital, Perambalur, IND

**Keywords:** womac index, risk factor, disability, screening tool, elderly population, osteoarthritis

## Abstract

Background: Osteoarthritis (OA) is a degenerative joint disease that occurs resulting from tear and progressive loss of articular cartilage. It is one of the leading causes of disability in elderly people. This study aims to assess the risk of OA and the ability to perform activities of daily living (ADL) independently among the geriatric population using the Western Ontario and McMaster Universities Arthritis Index (WOMAC) scale and Katz Index of Independence in Activities of Daily Living (Katz ADL) scale, respectively.

Methods: This cross-sectional study was conducted among the geriatric population in Perambalur district, Tamil Nadu from November 2022 to January 2023. Around 415 geriatric populations above 60 years of age were included by using a simple random sampling method. A semi-structured questionnaire was used to collect sociodemographic profiles, personal and medical details, OA risk (WOMAC), and ADL (Katz ADL scale). Descriptive statistics and the chi-square test were used to investigate the relationship between sociodemographic characteristics and the Katz ADL scale and the WOMAC index score for assessing OA risk.

Results: The mean age of participants was 69.62 ± 6.86 years. The mean ± SD score for the WOMAC scale and the Katz ADL scale among the geriatric population was 20.997 ± 14.69 and 4.821 ± 2.37, respectively. The OA risk among the geriatric population using the WOMAC scale was low in 98 (23.6%), moderate in 216 (52%), and high risk in 101 (24.3%) participants. Using the Katz ADL scale, 332 (80%) participants were found to be independent and 83 (20%) were dependent. The high-risk factors for developing OA were age ≥ 70 years, female sex, living in a rural area, employment status, Muslim religion, the habit of betel nut chewing, diabetes mellitus, hypertension, thyroid diseases, family history of knee OA, history of previous surgery, and ADL, significantly associated with WOMAC (p < 0.05).

Conclusion: As per the WOMAC scale, approximately 24.3% of the geriatric population is at high risk of developing OA and 20% of them are dependent on performing ADL as assessed using the Katz ADL scale. The WOMAC and Katz ADL scales are simple questionnaire-based screening tools used to detect high-risk individuals for OA at an early stage in the geriatric population.

## Introduction

Osteoarthritis (OA), also known as degenerative joint disease, of the knee is brought on by wear and strain as well as the progressive loss of articular cartilage. Most of the older age group is affected by it [1]. It is one of the main reasons why elderly people have lower limb disabilities. Limitations are imposed on the activity, which has an impact on social interactions, self-esteem, emotional health, and quality of life, especially in older people. In 2000, there were 6.091 billion elderly people worldwide. By 2025, that number is projected to increase to 8.039 billion [2]. The United Nations Population Division predicts that 34% of Indians will be above the age of 50 years by 2050. The percentage of people aged 65 years and older is projected to rise from 5% to 14% between 2010 and 2050, while the percentage of those aged 80 years and older will triple from 1% to 3% [3].

OA is indeed a common rheumatologic problem worldwide, and its prevalence varies depending on factors such as age, gender, and geography. In India, the prevalence of OA is estimated to be between 22% and 39%, with a higher prevalence in women and older adults [4].

It was discovered that women were more likely than males to have knee OA and that the prevalence was higher in rural than in metropolitan areas. Due to the high percentage of people who live in rural areas and work in jobs that require heavy physical labour, cultural practices such as sitting cross-legged and squatting, using Indian toilets, and walking without proper footwear may also be risk factors in India and contribute to the higher prevalence of knee OA in our nation [5,6].

Over a 12-year period, diabetes risk is 32% higher in people with OA [7]. Obesity and advanced age are risk factors for both OA and diabetes. OA may make it more difficult to exercise and reduce weight. The strongest modifiable risk factor for the onset of OA in the knee is also thought to be obesity. It has also been linked to a greater incidence of impairment [7].

OA screening needs various radiological and blood investigations to correlate with their clinical symptoms. But in India, not many people have access to or economic resources for that and many are unaware of their condition. In many cases, it may require frequent follow-up, medical therapy, and sometimes replacement surgeries, which are expensive and inaccessible to many patients. Hence early diagnosis and treatment remain the key to the management of OA [4,8].

Estimating prevalence in the geriatric population requires the tools that allow the investigators to screen a large sample drawn from the geriatric population to identify those individuals most likely to have the disease. The tool used in this study is the screening questionnaire. The two scales used in this questionnaire are the Western Ontario and McMaster Universities Arthritis Index (WOMAC) scale and the Katz Index of Independence in Activities of Daily Living (Katz ADL) scale [9,10].

This study aims to assess the risk of OA among the geriatric population using the WOMAC scale and assess the ability to perform activities of daily living (ADL) independently using the Katz ADL scale and determine the risk factors associated with OA among the geriatric population.

## Materials and methods

Study design and setting

This cross-sectional study was conducted among the geriatric population in Perambalur district, Tamil Nadu from November 2022 to January 2023.

Ethical consideration

Institutional Human Ethics Committee approval (Dhanalakshmi Srinivasan Medical College & Hospital; IECHS/IRCHS no.: 249; dated: November 11, 2022) was obtained before the start of the study and informed consent was taken before the start of the study.

Study participants

The participants aged above 60 years of both sexes were included. Participants who had a known diagnosis of OA and were bedridden were excluded from the study.

Sample size and sampling technique

According to Sathiyanarayanan et al.'s study [8], considering the prevalence of high risk for OA is 19.4%, the final estimated sample size with a 95% confidence interval and a 4% allowable error was calculated by using the following formula: n = 3.84 * P * Q/d^2^ (P = 19.4, Q = 80.6 (100-19.4), d = 4). The approximate sample size was 375. With a non-response rate of 10%, the expected final sample size was 413. A total of 415 samples were collected by using simple random sampling in the Perambalur district.

Data collection tool and technique

After getting the Institutional Ethics Committee's approval and informed consent from the participants, a pre-tested, semi-structured questionnaire was used to collect data. The questionnaire had four parts.

The first section included sociodemographic information, including age, sex, place of residence, education, income, and employment status. The modified BG Prasad scale was used to categorize socioeconomic status in the study population [11].

The second section included BMI as underweight (<18.5), normal (18.5-24.9), overweight (25-29.9), and obese (>30) [12], personal habits (alcohol consumption, smoking, betel nut chewing), and comorbidities, including diabetes mellitus, hypertension, thyroid disorders, cardiovascular disorders, epilepsy, trauma, asthma, chronic kidney disease, psychiatric illness, and a history of OA in the family and previous history of surgery.

The third section included the standard questionnaire (WOMAC) of 24 items to assess the risk of OA. The fourth section included the standard questionnaire (Katz ADL scale) of six items to assess ADL.

Measurements

WOMAC

Numerous studies employ the WOMAC scale to assess hip and knee OA [9]. The 24 items on the self-administered questionnaire are broken down into three subscales: pain (five items): whether walking, climbing stairs, resting in bed, sitting, or standing; two instances of stiffness: when you first wake up and later in the day; and physical function (17 items): utilizing stairs, getting up from a chair, standing, bending, walking, shopping, putting on/taking off socks, rising from a lying position, bathing, sitting, using the toilet, and heavy and light housework. The test questions are scored on a scale of 0-4, which correspond to none (0), mild (1), moderate (2), severe (3), and extreme (4). The scores for each subscale are summed up, with a possible score range of 0-20 for pain, 0-8 for stiffness, and 0-68 for physical function. Higher scores on the WOMAC indicate worse pain, stiffness, and functional limitations. The total score for the WOMAC is 0-59 (possible cut-off 0-96), which is categorized as low risk (<11), average risk (11-30), and high risk (>30). This categorization is based on the percentiles where more than the 75th percentile is taken as high risk, the 25th to 75th as average risk, and less than the 25th percentile is taken as low risk.

Katz ADL

The most suitable tool to evaluate functional status as a measurement of the client's capacity to carry out ADL independently is the Katz Index of Independence in Activities of Daily Living, also known as the Katz ADL. The Katz ADL gauges a person's independence in basic daily living activities (ADL) [10]. An effective way to evaluate the health status of older adults is through their functional ability. An objective assessment, which provides objective data, helps indicate a decline or improvement in health status, allowing the physiotherapist to plan and intervene appropriately. The Index ranks adequacy of performance in six functions: bathing, dressing, toileting, transferring, continence, and feeding. One point means the person is independent; zero points means the person requires supervision, direction, personal assistance, or total care. A total score of 5 or 6 is independent and a score <5 is dependent.

Statistical analysis

Data were entered on a Microsoft Excel sheet (Microsoft Corporation, Redmond, WA) and analysed using statistical software SPSS version 26 (IBM Corp., Armonk, NY). Frequency and percentage were used to represent qualitative data, while mean and standard deviation were used to represent quantitative data. The chi-square test was used to check the association between sociodemographic characteristics and risk factors to WOMAC OA risk index and p < 0.05 was considered statistically significant.

## Results

A total of 415 samples were collected from the geriatric population residing in the Perambalur district of Tamil Nadu, India. The mean age of the participants was 69.62 ± 6.86 years. Most of the geriatric population belonged to those aged <70 years (244, 58.8%), followed by those aged ≥70 years (171, 41.2%), and residing in rural areas (352, 84.8%). Among them, more than half of the participants were illiterate (282, 68%) and unemployed (262, 63.1%). The majority were married (298, 71.8%) and belonged to the Hindu religion (369, 88.9%), and had class 3 socioeconomic status followed by class 2 (Table 1).

**Table 1:** Sociodemographic characteristics of study participants (n = 415)

Sociodemographic characteristics		n (%)
Place of residence	Rural	352 (84.8%)
	Urban	63 (15.2%)
Age group (in years)	<70	244 (58.8%)
	≥70	171 (41.2%)
Gender	Male	191 (46%)
	Female	224 (54%)
Education	Illiterate	282 (68%)
	Literate	133 (32%)
Employment status	Employed	153 (36.9%)
	Unemployed	262 (63.1%)
Marital status	Married	298 (71.8%)
	Spouse diseased/separated & unmarried	117 (28.2%)
Religion	Hindu	369 (88.9%)
	Muslim	16 (3.9%)
	Christian	30 (7.2%)
Socioeconomic status	Class 1	53 (12.8%)
	Class 2	152 (36.6%)
	Class 3	158 (38.1%)
	Class 4	37 (8.9%)
	Class 5	15 (3.6%)
Body mass index (BMI)	Underweight	16 (3.9%)
	Normal	301 (72.5%)
	Overweight	96 (23.1%)
	Obese	2 (0.5%)

The mean ± SD of the WOMAC score, which is used for screening of OA risk, and the Katz ADL scale, which is used for assessing ADL among the geriatric population, was 20.997 ± 14.69 and 4.821 ± 2.37, respectively (Table 2).

**Table 2:** Osteoarthritis screening tool scores and Katz ADL score among the study population (n = 415) WOMAC: Western Ontario and McMaster Universities Arthritis Index; Katz ADL: Katz Index of Independence in Activities of Daily Living.

Screening tool		Mean ± SD	Minimum	Maximum	Possible cutoff
Domains of WOMAC score	Pain	4.42 ± 3.15	0	15	0-20
	Stiffness	0.64 ± 0.95	0	4	0-8
	Physical function	15.93 ± 11.66	0	51	0-68
Total WOMAC score (pain + stiffness + physical function)		20.99 ± 14.69	0	59	0-96
Katz ADL score		4.82 ± 2.37	0	6	0-6

**Figure 1:**
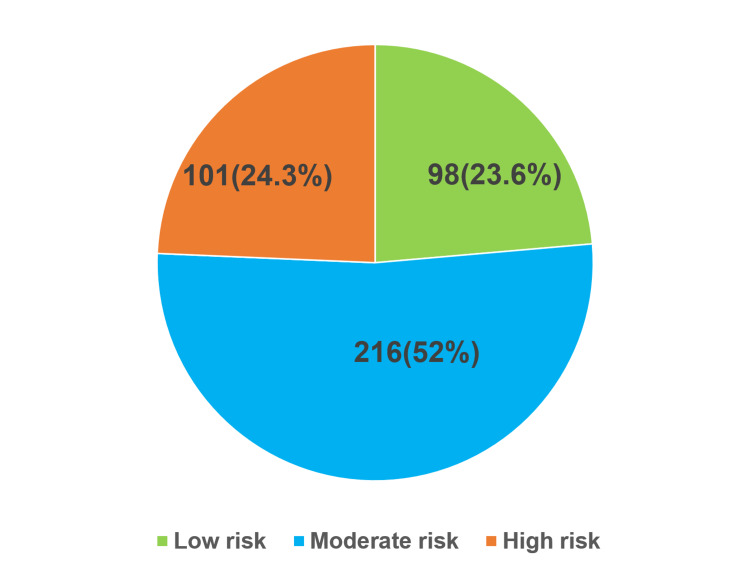
Categorization of osteoarthritis risk using the WOMAC scale (n = 415) WOMAC: Western Ontario and McMaster Universities Arthritis Index.

Figure 1 shows the categorization of OA risk among the geriatric population using the WOMAC scale as low risk (98, 23.6%), moderate risk (216, 52%), and high risk (101, 24.3%).

Tables 3, 4 show the association between risk factors (sociodemographic and personal details) and the WOMAC score. Participants with age ≥ 70 years (47, 27.5%), females (65, 29%), participants residing in rural areas (99, 28.1%), employed (53, 34.6%), and participants following the Islamic religion (5, 31.3%) are at high risk of developing OA compared to their counterparts and such differences are statistically significant (p = 0.009, p = 0.045, p < 0.001, p = 0.001, and p = 0.007, respectively). Likewise, participants who were having increased weight/obese (44, 44.4%), had the habit of betel nut chewing (44, 44.4%), had diabetes mellitus (67, 37.2%), hypertension (38, 38%), thyroid diseases (8, 72.7%), family history of knee OA (2, 100%), and history of previous surgery (16, 39%) were found to be at high risk for developing OA, which is statistically significant (p < 0.05).

**Table 3:** Association between sociodemographic characteristics and WOMAC among the study population (n = 415) The chi-square test was applied. * P-value < 0.05 is statistically significant. WOMAC: Western Ontario and McMaster Universities Arthritis Index.

Characteristics		Osteoarthritis risk (WOMAC scale)			p-value
		Low	Moderate	High	
Age (in years)	<70	48 (19.7%)	142 (58.2%)	54 (22.1%)	0.009^*^
	≥70	50 (29.2%)	74 (43.3%)	47 (27.5%)	
Gender	Male	51 (26.7%)	104 (54.5%)	36 (18.8%)	0.045^*^
	Female	47 (21%)	112 (50%)	65 (29%)	
Place of residence	Rural	80 (22.7%)	173 (49.1%)	99 (28.1%)	<0.001^*^
	Urban	18 (28.6%)	43 (68.3%)	2 (3.2%)	
Education	Illiterate	66 (23.4%)	144 (51.1%)	72 (25.5%)	0.706
	Literate	32 (24.1%)	72 (54.1%)	29 (21.8%)	
Employment status	Employed	35 (22.9%)	65 (42.5%)	53 (34.6%)	0.001^*^
	Unemployed	63 (24%)	151 (57.6%)	48 (18.3%)	
Marital status	Married	70 (23.5%)	161 (54%)	67 (22.5%)	0.186
	Spouse diseased/separated/unmarried	28 (25.2%)	55 (48.5%)	34 (26.2%)	
Religion	Hindu	80 (21.7%)	200 (54.2%)	89 (24.1%)	0.007^*^
	Muslim	3 (18.8%)	8 (50%)	5 (31.3%)	
	Christian	15 (50%)	8 (26.7%)	7 (23.3%)	
Socioeconomic status	Class 1	18 (34%)	27 (50.9%)	8 (15.1%)	0.518
	Class 2	33 (21.7%)	82 (53.9%)	37 (24.3%)	
	Class 3	38 (24.1%)	81 (51.3%)	39 (24.7%)	
	Class 4	6 (16.2%)	19 (51.4%)	12 (32.4%)	
	Class 5	3 (20%)	7 (46.7%)	5 (33.3%)	

**Table 4:** Association between risk factors for osteoarthritis and WOMAC among the study population (n = 415) The chi-square test was applied. * P-value < 0.05 is statistically significant. WOMAC: Western Ontario and McMaster Universities Arthritis Index.

Risk factors		Osteoarthritis risk (WOMAC)			p-value
		Low	Moderate	High	
Body mass index (BMI)	Underweight	9 (56.3%)	6 (37.5%)	1 (6.3%)	<0.001^*^
	Normal	80 (26.6%)	165 (54.8%)	56 (18.6%)	
	Overweight/obese	9 (9.2%)	45 (45.9%)	44 (44.9%)	
Smoking	Yes	7 (16.7%)	24 (57.1%)	11 (26.2%)	0.534
	No	91 (24.4%)	192 (51.5%)	90 (24.1%)	
Alcohol intake	Yes	16 (19%)	47 (56%)	21 (25%)	0.533
	No	82 (24.8%)	169 (51.1%)	80 (24.2%)	
Betel nut chewing	Yes	10 (10.1%)	45 (45.5%)	44 (44.4%)	<0.001^*^
	No	88 (27.8%)	171 (54.1%)	57 (18%)	
Diabetes mellitus	Yes	26 (14.4%)	87 (48.3%)	67 (37.2%)	<0.001^*^
	No	72 (30.6%)	129 (54.9%)	34 (14.5%)	
Hypertension	Yes	15 (15%)	47 (47%)	38 (38%)	0.001^*^
	No	83 (26.3%)	169 (53.7%)	63 (20%)	
Cardiovascular disease	Yes	1 (11.1%)	4 (44.4%)	4 (44.4%)	0.326
	No	97 (23.6%)	212 (52.2%)	97 (23.9%)	
Thyroid disease	Yes	2 (18.2%)	1 (9.1%)	8 (72.7%)	0.001^*^
	No	96 (23.8%)	215 (53.2%)	93 (23%)	
Trauma	Yes	1 (11.1%)	3 (33.3%)	5 (55.6%)	0.086
	No	97 (23.9%)	213 (52.5%)	96 (23.6%)	
Family history of osteoarthritis	Yes	-	-	2 (100%)	0.044^*^
	No	98 (23.7%)	216 (52.3%)	99 (24%)	
Previous surgery	Yes	12 (29.3%)	13 (31.7%)	16 (39%)	0.016^*^
	No	86 (23%)	203 (54.3%)	85 (22.7%)	
Hysterectomy (n = 41)	Yes	4 (50%)	1 (12.5%)	3 (37.5%)	0.270
	No	8 (24.2%)	12 (36.4%)	13 (39.4%)	

**Figure 2:**
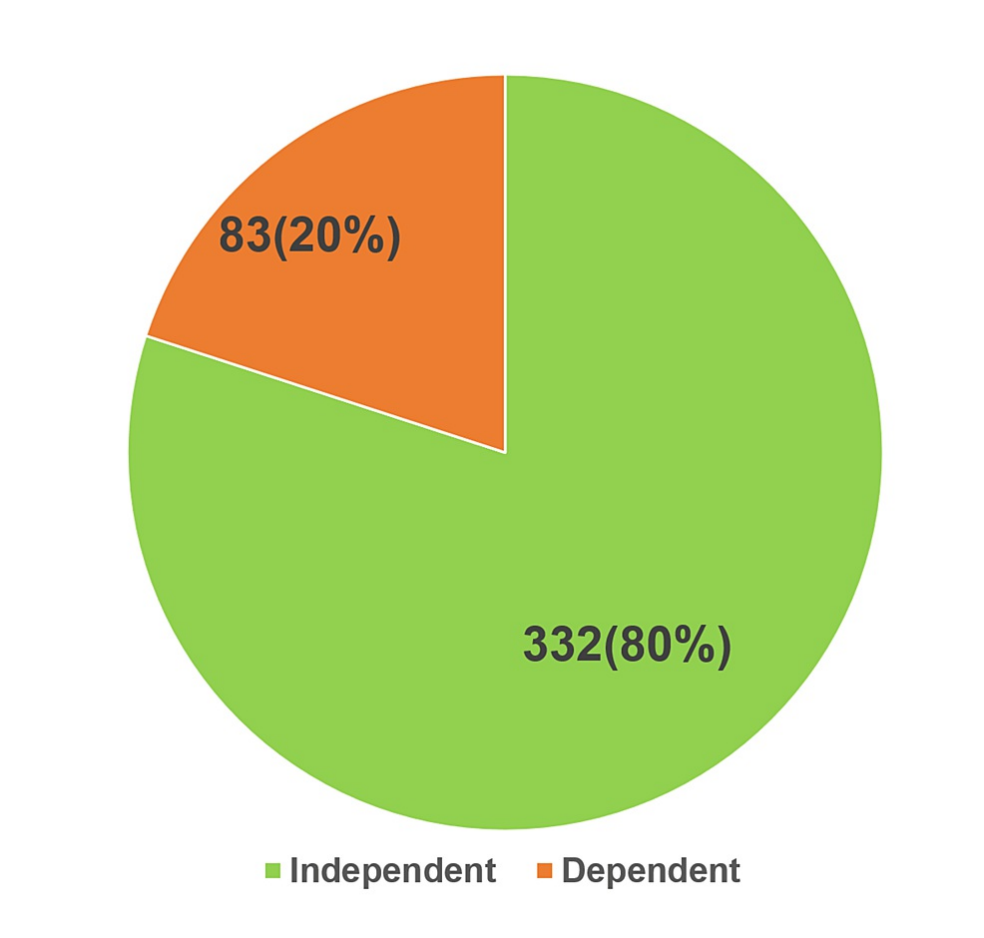
Categorization of activities of daily living using the Katz ADL scale Katz ADL: Katz Index of Independence in Activities of Daily Living.

Figure 2 shows the categorization of ADL using the Katz ADL scale, in which more than three-fourths of participants were independent (332, 80%) and 83 (20%) were dependent.

Table 5 depicts an association between OA risk and ADL. Around 69 (83.1%) dependent participants were at moderate risk of developing OA when compared to independent individuals.

**Table 5:** Association between WOMAC score and Katz ADL index The chi-square test was applied. * P-value < 0.05 is statistically significant. WOMAC: Western Ontario and McMaster Universities Arthritis Index; Katz ADL: Katz Index of Independence in Activities of Daily Living.

Katz ADL score	WOMAC score			p-value
	Low	Moderate	High	
Independent	86 (25.9%)	147 (44.3%)	99 (26.8%)	<0.001^*^
Dependent	12 (14.5%)	69 (83.1%)	2 (2.4%)	

## Discussion

In this study, people of age above 60 years in both males and females were considered. WOMAC is used for finding the risk of the development of OA among the geriatric population residing in both rural and urban areas. The Katz ADL index was used for finding the level of dependency in individuals and then their association was established. Numerous risk factors for OA were studied to find the risk of OA development.

In the current study, OA risk among the geriatric population using the WOMAC scale was low risk in 98 (23.6%), moderate risk in 216 (52%), and high risk in 101 (24.3%) participants. Similarly, a study done in Tamil Nadu [8] showed similar findings, and a study in Karnataka by Radha and Gangadhar [13] and in Gujarat by Abhinav Kotak [14] showed the majority of participants were at moderate risk of OA.

The present study revealed that the geriatric population aged ≥70 years (47, 27.5%), female sex (65, 29%), and overweight and obese participants (44, 44.9%) were at higher risk of developing OA when compared to participants aged <70 years. Similarly, a study done in India [8] showed that with increasing age, females and overweight and obese people were at higher risk of developing OA when compared to their counterparts. These findings are also consistent with studies done by Fang et al. [15] and Zhang et al. [16] and a literature review done by Heidari [5]. Therefore, different strategies may be required in the evaluation and management of males and females with knee OA to improve their quality of life.

In this study, participants residing in rural areas (99, 28.1%) were at higher risk of developing OA when compared to the urban population. Current study findings were consistent with Jaiswal et al. in Haryana [17] and Heidari [5]. This may be due to a lack of awareness and low literacy level, low healthcare system utilization, and following self-medication and heavy workload like lifting weights etc. among the rural population, which in turn causes risk of OA.

In the present study, the participants having higher BMI were at high risk of developing OA, which is statistically significant (p < 0.001). Similarly, studies revealed that people with higher BMI are at risk of developing OA [18,19]. Obesity is considered a significant modifiable risk factor for the onset of knee OA. Excess body weight places increased mechanical stress on the joints, particularly the weight-bearing joints like the knees, leading to accelerated wear and tear of the joint cartilage. The increased load on the joint can contribute to the development and progression of OA. In fact, studies have shown that obesity is strongly associated with knee OA and weight loss has been shown to reduce the risk and progression of knee OA.

However, the presence of OA can pose challenges to exercise and weight reduction efforts. The pain and functional limitations associated with OA may make it more difficult for individuals to engage in physical activity and maintain a healthy weight. This can create a vicious cycle where the difficulty in exercising and losing weight may further contribute to joint deterioration and increased impairment.

In the current study, comorbidities such as hypertension (38, 38%), diabetes (67, 37.2%), and thyroid disorder (8, 72.7%) were found to be high-risk factors for developing OA. Similarly, a study done in China by Liu et al. [20] showed that multiple metabolic disorders are strongly associated with OA. Of people with a previous history of surgeries, 16 (39%) showed a significant association with the development of OA. Similarly, a retrospective review study done in Missouri during 2005-2009 revealed that previous history of surgery leads to a high risk of knee OA [21]. Therefore, for a person with a history of previous surgery like total knee or hip replacement, there are possibilities of less use of the limb, which in turn causes a risk of OA.

In the present study, there was no significant association of OA with hysterectomy. Similarly, there are studies that showed hysterectomy as a risk factor suggesting there may be a modest association of hysterectomy with the onset of knee OA, although results were non-significant [18]. We found a significant association between a family history of OA (2, 100%) and WOMAC scores. Similarly, a study done in Haryana by Jaiswal et al. [17] found that family history is one of the independent risk factors for OA.

Strength and limitations

The strength of the present study lies in the large sample size of 415 individuals, which allows for a comprehensive investigation of the development of OA among them. A large sample size is generally desirable as it can increase the statistical power of the study and enhance the reliability of the findings.

However, there are some limitations to consider. Although a large number of individuals were included in the study, the majority of them were illiterate and unaware of their situation. This poses a challenge when using a questionnaire-based tool for data collection, as illiteracy and lack of awareness may affect the participant's ability to accurately respond to the questions. This limitation could potentially introduce measurement errors and affect the validity of the results.

Additionally, the study did not utilize radiographic analysis, which is a commonly used method for identifying high-risk individuals for OA. The reasons for not utilizing radiographic analysis were mentioned as economic factors and resource unavailability. This limitation could have implications for the accuracy and precision of identifying individuals at high risk for OA, as radiographic analysis is considered more objective and reliable in detecting structural changes associated with OA.

Consequently, the generalizability of the results may be limited. The study sample predominantly consists of illiterate individuals who may not be representative of the broader population. Therefore, caution should be exercised when applying the findings of this study to other populations with different literacy levels and socioeconomic backgrounds.

To address these limitations and further advance research in this area, future studies should consider incorporating intervention studies or qualitative research methods. Intervention studies can provide valuable insights into the effectiveness of different interventions or treatments for OA, while qualitative studies can explore the lived experiences and perceptions of individuals with OA, offering a deeper understanding of the condition.

In summary, while the large sample size is a notable strength of the present study, the limitations related to the literacy level of participants, the exclusion of radiographic analysis, and the generalizability of the findings highlight the need for further research using alternative methods and diverse populations.

## Conclusions

Around one-fourth of the 101 (24.3%) geriatric population are at high risk of developing OA. Around 83 (20%) participants were dependent on doing the activities of daily living assessed by using Katz ADL. Participants aged ≥70 years, females, participants who resided in a rural area, had employment, followed the Islamic religion, had increased BMI, had the habit of betel nut chewing, had diabetes mellitus, hypertension, thyroid diseases, family history of knee OA, and history of previous surgery, and who were dependent on others for ADL were found to be at high risk for developing OA.

OA is one of the most prevalent chronic diseases among the geriatric population and early diagnosis and treatment remain a key factor in reducing the morbidity of the disease. In developing and resource-constraint countries like India, a simple questionnaire-based tool to detect high-risk individuals for knee OA at an early stage is very useful at this moment. WOMAC is usually used to evaluate the effectiveness of treatment for OA patients and can be used as a screening tool.

## References

[1] Kurtaiş Y, Oztuna D, Küçükdeveci AA, Kutlay S, Hafiz M, Tennant A (2011). Reliability, construct validity and measurement potential of the ICF comprehensive core set for osteoarthritis. BMC Musculoskelet Disord.

[2] Rajan SI, Sarma PS, Mishra US (2003). Demography of Indian aging, 2001-2051. J Aging Soc Policy.

[3] Fransen M, Bridgett L, March L, Hoy D, Penserga E, Brooks P (2011). The epidemiology of osteoarthritis in Asia. Int J Rheum Dis.

[4] Pal CP, Singh P, Chaturvedi S, Pruthi KK, Vij A (2016). Epidemiology of knee osteoarthritis in India and related factors. Indian J Orthop.

[5] Heidari B (2011). Knee osteoarthritis prevalence, risk factors, pathogenesis and features: part I. Caspian J Intern Med.

[6] Haq SA, Darmawan J, Islam MN (2008). Incidence of musculoskeletal pain and rheumatic disorders in a Bangladeshi rural community: a WHO-APLAR-COPCORD study. Int J Rheum Dis.

[7] Piva SR, Susko AM, Khoja SS, Josbeno DA, Fitzgerald GK, Toledo FG (2015). Links between osteoarthritis and diabetes: implications for management from a physical activity perspective. Clin Geriatr Med.

[8] Sathiyanarayanan S, Shankar S, Padmini SK (2017). Usefulness of WOMAC index as a screening tool for knee osteoarthritis among patients attending a rural health care center in Tamil Nadu. Int J Community Med Public Health.

[9] Bellamy N, Buchanan WW, Goldsmith CH, Campbell J, Stitt LW (1988). Validation study of WOMAC: a health status instrument for measuring clinically important patient relevant outcomes to antirheumatic drug therapy in patients with osteoarthritis of the hip or knee. J Rheumatol.

[10] Sharifi F, Alizadeh-Khoei M, Saghebi H (2018). Validation study of ADL-Katz scale in the Iranian elderly nursing homes. Ageing Int.

[11] Debnath D, Kakkar R (2020). Modified BG Prasad socio-economic classification, updated - 2020. Indian J Community Health.

[12] Nuttall FQ (2015). Body mass index: obesity, BMI, and health: a critical review. Nutr Today.

[13] Radha MS, Gangadhar MR (2015). Prevalence of knee osteoarthritis patients in Mysore city, Karnataka. Int J Recent Sci Res.

[14] Kotak A (2016). Prevalence of knee osteoarthritis patients in Bhuj, Kutch, Gujarat, India - a cross-sectional study. Int J Sci Res.

[15] Fang WH, Huang GS, Chang HF (2015). Gender differences between WOMAC index scores, health-related quality of life and physical performance in an elderly Taiwanese population with knee osteoarthritis. BMJ Open.

[16] Zhang Y, Jordan JM (2010). Epidemiology of osteoarthritis. Clin Geriatr Med.

[17] Jaiswal A, Goswami K, Haldar P, Salve HR, Singh U (2021). Prevalence of knee osteoarthritis, its determinants, and impact on the quality of life in elderly persons in rural Ballabgarh, Haryana. J Family Med Prim Care.

[18] Blagojevic M, Jinks C, Jeffery A, Jordan KP (2010). Risk factors for onset of osteoarthritis of the knee in older adults: a systematic review and meta-analysis. Osteoarthritis Cartilage.

[19] Cooper DJ, Scammell BE, Batt ME, Palmer D (2018). Factors associated with pain and osteoarthritis at the hip and knee in Great Britain's Olympians: a cross-sectional study. Br J Sports Med.

[20] Liu Y, Zhang H, Liang N (2016). Prevalence and associated factors of knee osteoarthritis in a rural Chinese adult population: an epidemiological survey. BMC Public Health.

[21] Smith MV, Nepple JJ, Wright RW, Matava MJ, Brophy RH (2017). Knee osteoarthritis is associated with previous meniscus and anterior cruciate ligament surgery among elite college American football athletes. Sports Health.

